# Cellular Uptake of Plain and SPION-Modified Microbubbles for Potential Use in Molecular Imaging

**DOI:** 10.1007/s12195-017-0504-9

**Published:** 2017-08-10

**Authors:** Mona Ahmed, Barbara Cerroni, Anton Razuvaev, Johan Härmark, Gaio Paradossi, Kenneth Caidahl, Björn Gustafsson

**Affiliations:** 1 Department of Molecular Medicine and Surgery, Karolinska Institutet, Stockholm, Sweden; 2 Department of Chemical Sciences and Technologies, University of Rome Tor Vergata, Rome, Italy; 3 School of Technology and Health, KTH Royal Institute of Technology, Stockholm, Sweden; 4 Department of Biosciences and Nutrition, Karolinska Institutet, Huddinge, Sweden; 5 Department of Clinical Physiology C8:27, Karolinska University Hospital, Stockholm, SE-171 76 Sweden

**Keywords:** Polyvinyl-alcohol, Macrophages, Endothelial cells, Interactions, *In vitro*

## Abstract

**Introduction:**

Both diagnostic ultrasound (US) and magnetic resonance imaging (MRI) accuracy can be improved by using contrast enhancement. For US gas-filled microbubbles (MBs) or silica nanoparticles (SiNPs), and for MRI superparamagnetic or paramagnetic agents, contribute to this. However, interactions of MBs with the vascular wall and cells are not fully known for all contrast media.

**Methods:**

We studied the* in vitro* interactions between three types of non-targeted air-filled MBs with a polyvinyl-alcohol shell and murine macrophages or endothelial cells. The three MB types were plain MBs and two types that were labelled (internally and externally) with superparamagnetic iron oxide nanoparticles (SPIONs) for US/MRI bimodality. Cells were incubated with MBs and imaged by microscopy to evaluate uptake and adhesion. Interactions were quantified and the MB internalization was confirmed by fluorescence quenching of non-internalized MBs.

**Results:**

Macrophages internalized each MB type within different time frames: plain MBs 6 h, externally labelled MBs 25 min and internally labelled MBs 2 h. An average of 0.14 externally labelled MBs per cell were internalized after 30 min and 1.34 after 2 h; which was 113% more MBs than the number of internalized internally labelled MBs. The macrophages engulfed these three differently modified new MBs at various rate, whereas endothelial cells did not engulf MBs.

**Conclusions:**

Polyvinyl-alcohol MBs are not taken up by endothelial cells. The MB uptake by macrophages is promoted by SPION labelling, in particular external such, which may be important for macrophage targeting.

## Introduction

Inflammatory processes are involved in many diseases,^3^,^38^ and are sometimes easily recognized. However, there are several conditions in which inflammation is more difficult to diagnose, such as various types of cancer,^3^,^13^ and vascular wall inflammation and repair in cardiovascular diseases (CVD) e.g., atherosclerosis.^21^,^33^,^51^ Molecular imaging techniques have become important for detection, and with possibly for targeted treatment, of inflammation in CVD.^27^,^52^,^61^ The use of hybrid imaging methods, such as single-photon emission computed tomography (SPECT)/computed tomography (CT), positron emission tomography (PET)/CT, and PET/magnetic resonance imaging (MRI), is increasing. With MRI, the use of contrast considerably increases the diagnostic yield for various diseases.^41^ However, ultrasound (US) remains the most widely used diagnostic imaging tool, and research about combining US with other imaging techniques is ongoing.

Currently, the standard for clinical atherosclerosis imaging is based on visualization of vessel stenosis and plaque morphology.^15^ However, obtaining important biological information at the cellular level can be limited, and further work is needed to improve the visualization of vessels and cellular activities. The cellular composition of vulnerable plaques is different from that of stable plaques, but recent reports show that this might be achieved by using well-designed contrast agents and molecular imaging techniques.^2^,^14^,^63^

With the growing interest in molecular imaging, including the need to detect atherosclerotic inflammation,^6^,^16^,^26^,^43^,^52^,^59^,^61^ there is a demand for new imaging probes to be used in the various imaging modalities currently available. New probes would need to be specifically targeted to relevant cells in order to be clinically useful. In this context, the cells of interest are mainly macrophages, which are involved in inflammatory processes such as atherosclerosis, and endothelial cells, which outline all the vasculature and play a key role in primary vascular defense.

The synthetic organic polymer polyvinyl alcohol (PVA) is a biocompatible material for drug carriers and has shown potential for various medical applications in both diagnostics and theranostics.^10^,^39^,^58^,^60^ PVA enhances cellular interactions and is used in the form of nano- or micro-particles as coating for nanoparticles and hydrogels.^5^,^18^,^25^

The combination of US and MRI contrast agents in one bi-modal probe remain scarce^36^ but recent publications have shown an increasing interest for this combination of modalities.^20^,^24^,^48^

So in an effort to develop new US contrast agents that are specifically recognized by inflammatory cell markers, a previously developed air-filled PVA microbubble (MB), 3 μm in size was further functionalized, within the scope of the European Commission’s 3MiCRON project, for potential MRI by conjugation of superparamagnetic iron oxide nanoparticles (SPIONs).^9^,^47^ The different conjugations of SPIONs to plain MBs, as well as the physical, magnetic, and US-imaging properties of these MBs were recently reported.^7^,^22^,^23^,^28^,^29^,^31^,^32^,^49^,^54^ The* in vitro* T_2_*-relaxivity, biodistribution, and* in vivo* pharmacokinetics of one of these MBs were evaluated using histology and MRI,^4^ and just recently a study by Sciallero* et al*. fully characterizing the US and MR imaging properties of these bubbles was published.^53^ Micro-devices similar to these have also been identified useful within fields as imaging and theranostics and are reported recently.^8^,^56^,^64^

In this study, we used* in vitro* cell models to evaluate the interactions of two cell types, namely macrophages and endothelial cells, with plain PVA MBs and two types of SPION-conjugated MBs. Our study provided information about the ability of these cells to internalize, or attach to, these MBs.

## Materials and Methods

**Figure 1:**
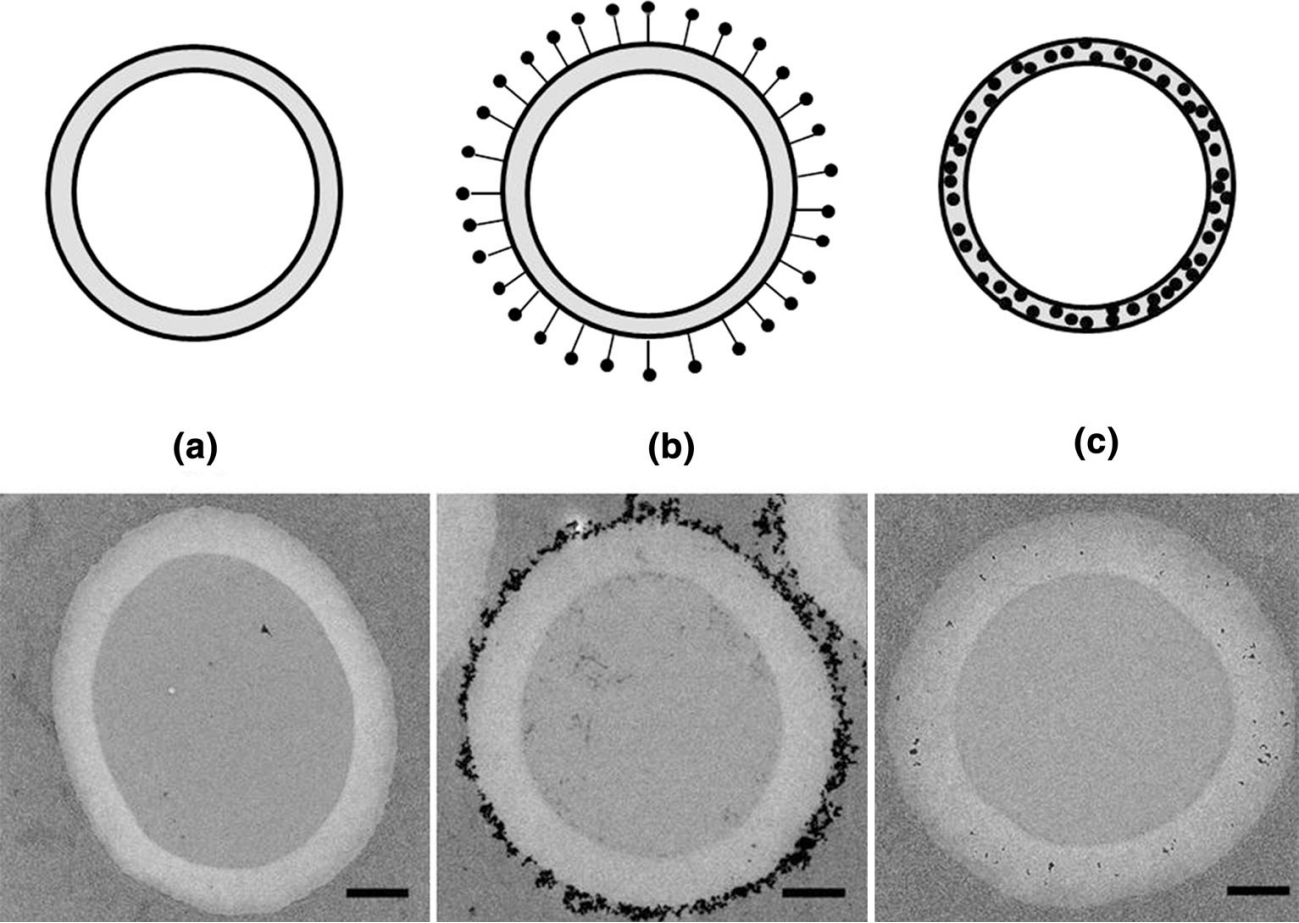
Upper panel: Schematic of the three types of bubbles used: (a) plain; (b) type A MBs with external SPIONs; and (c) type B MBs with internal SPIONs. Black dots represent SPIONs. Lower panel: Transmission electron microscope images of the three different types of MBs: (a) plain MBs; (b) type A MBs; and (c) type B MBs. Scale bar represents 500 nm.

All the* in vitro* experiments were performed using two mouse cell lines, the RAW264.7 monocytes/macrophages and MyEnd^+/+^ microvascular myocardial endothelial cells. The cell cultures were established in cell culture flasks, μ-slides (Ibidi^®^, Munich, Germany), or OptiCell™ (OC) chambers (Thermo Scientific, Waltham, MA, USA). For time-lapse studies, a SmartSlide™-6 micro-incubator (WaferGen Biosystems, Freemont, CA, USA) was used. The cells were cultured in Dulbecco’s modified Eagle’s medium with 1 g l^−1^ glucose and supplemented with 1% l-glutamine, 0.4% penicillin–streptomycin, 1% sodium pyruvate and 10% fetal bovine serum (Thermo Scientific). All incubations were performed in an incubator at 37 °C and 5% CO_2_. Several different experiments were performed in which the cells were incubated with different types of MBs: plain PVA MBs and two types (types A and B) of MBs with SPIONs attached. Type A MBs were bubbles with SPIONs attached to the surface with a chemical bond, and type B MBs had SPIONs physically embedded inside the PVA shell (Fig. 1). The interactions between the MBs and the cells were evaluated using conventional light microscopy and fluorescent/confocal microscopy.

### Microbubbles

The synthesis of plain PVA-based MBs has been reported.^9^ Briefly, sodium metaperiodate was added to an aqueous PVA solution (2% w/v) to obtain shorter PVA chains with terminal aldehyde groups.^9^ The acetalization reaction between these groups and the hydroxyl groups present in the polymer chains was performed under high-shear stirring (8000 rpm for 2 h, pH 5.5) in an Ultra-Turrax (IKA^®^-Werke GmbH & Co. KG, Staufen, Germany) homogenizer equipped with a Teflon tip at ambient temperature. MBs with an average size of 3 μm were produced. The remaining aldehyde groups on the MB surface after this cross-linking reaction were used for further modifications. Two different modifications to the MB shell were performed: the SPIONs were either covalently attached to the surface of the polymer MBs* via* reductive amination (type A MBs) or embedded in the PVA shell of the MB (type B MBs).^7^,^40^,^55^ MB concentration was determined by manual counting using a haemocytometer.

### Cell Cultures

#### Macrophages

The RAW264.7 mouse leukemic monocyte macrophage cell line was used. Cells were purchased from ATCC (Manassas, VA, USA) or Istituto Zooprofilattico della Lombardia e dell’Emilia-Romagna. This cell line was established from the ascites of a tumor induced in a male mouse by intraperitoneal injection of the Abelson murine leukemia virus. These cells have receptors for immunoglobulin and produce lysozyme.^50^ The RAW264.7 cells proliferated well in flasks, and most of the cells attached well and spread out evenly. Their cell morphology varied from oval to more rounded cells.

#### Endothelial Cells

MyEnd^+/+^ endothelial cells (murine microvascular myocardial endothelial cells, also named EC-VASP^+/+^ cells) were grown on a 0.2% gelatin matrix in flasks.^1^,^19^ The cells attached well to the flasks and proliferated well in the optimum milieu for these cells.

### RAW264.7 Incubated with Plain MBs

RAW264.7 cells were seeded in four OC chambers at a concentration of 150,000 cells ml^−1^ in each OC, yielding a surface density of 3 × 10^4^ cells cm^−2^ and a total of 1.5 × 10^6^ cells in each OC. The OCs were placed in the incubator with their A-side facing up, which allowed the cells to attach to the B-side. Images were taken with a light microscope after seeding the cells in the OCs and 18–24 h later. After 2 days of proliferation plain MBs, sterilized with UV-light, were added to the OCs containing the cells at three different final concentrations (10^5^, 10^6^, and 5 × 10^6^ ml^−1^) (named OC1, OC2, and OC3, respectively). One OC was kept as a control (named OC4) with only cells, and one OC received only MBs at a concentration of 10^6^ ml^−1^ (named OC5). The OCs were placed in the incubator horizontally with their cell surface on the B-side facing up, which allowed the floating MBs to interact with the cells. Images were taken of the cell cultures before and after 1, 19, 26, and 48 h incubation with MBs. After 48 h, the medium was changed to wash away loose MBs and new images were taken.

Uptake of plain fluorescent MBs by macrophages was also evaluated in a second study using confocal laser scanning microscopy (CLSM) (Nikon Eclipse Ti, Tokyo, Japan). After 6 h of incubation of 10^5^ RAW264.7 with 2 × 10^7^ rhodamine-labelled MBs, the cells were stained using phalloidin–fluorescein isothiocyanate (FITC), which specifically binds to F-actin, to induce green fluorescent labelling of the cytoskeleton. Phalloidin–FITC was solubilized in dimethyl sulfoxide to form a stock solution at a concentration of 0.1 mg ml^−1^. Cells were rinsed twice with phosphate-buffered saline (PBS) and then fixed with 3.7% formaldehyde in PBS for 10 min. After washing with PBS, the cells were permeabilized with 0.1% Triton-X 100 in PBS for 7 min. The specimens were washed with PBS and stained using a 50 μg ml^−1^ fluorescent phalloidin–FITC solution in PBS for 40 min at room temperature. The cells were rinsed with PBS twice and observed by CLSM. Green and red fluorescence was detected by exciting the samples at 488 and 543 nm with Ar ion (Spectra Physics, Santa Clara, CA, USA) and He–Ne (Melles Griot, Albuquerque, NM, USA) lasers, respectively. Image capture and processing were performed using Nikon EZ-C1 software (version 3.9).

### RAW264.7 and MyEnd^+/+^ Cells Incubated with SPION-Coated MBs and Evaluation of Internalized MBs

For the incubation of cells with MBs, Ibidi^®^ slides (μ-Slide VI^0.1^) were used. The cells (1.7 × 10^4^) were seeded in each Ibidi^®^ channel (height of channels, 0.1 mm) of a μ-Slide VI^0.1^ and placed upside down in the incubator to permit cell attachment in the ceiling of each channel. After 12 h, cells were incubated with FITC-labelled MBs and types A and B SPION–FITC-labelled^9^ MBs. About 3 × 10^5^ MBs (~15 MBs/cell) were then added, and the Ibidi^®^ slide was placed in the incubator in upright orientation for different incubation times. When positioned this way, the floating MBs could interact with cells attached at the top of the channel. After incubation (30 min or 2 h), the uptake of MBs by macrophages was observed using CLSM and quantified by manually counting MBs and cells in five to nine random regions-of-interest (ROIs) counting 50–300 cells per ROI. The cells were monitored in transmission mode and the FITC-labelled MBs were visualized by laser excitation at 488 nm. To discriminate between internalized and extracellular MBs, the MB suspension was replaced by 1.7 μl (total channel volume) of Trypan Blue (TB, 0.1 mg ml^−1^) in PBS per channel to quench the extracellular FITC fluorescence. Resulting in the quenching of external MBs and the non-quenching of internalized MBs. After 1 min, the cells were monitored using CLSM.

### Time-Lapse Study

For the time-lapse study, about 5.5 × 10^5^ macrophages were seeded in each well of a SmartSlide-6™ micro-incubator and incubated for 12 h to permit cell adhesion. About 1.1 × 10^7^ (20 MBs/cell) type A SPION–MBs were added to cells, and the SmartSlide-6™ was fitted on the automated microscope table. To impede the floating of the MBs the air in the MBs was replaced with PBS, briefly; keep 10^8^ MB ml^−1^ in a 45% ethanol solution for 3 days to transform MBs into microcapsules, then centrifuge for 10 min at 1000 rpm and wash with PBS three times The SmartSlide-6™ micro-incubator was temperature-controlled at 37°C and perfused with 5% CO_2_. In the time-lapse experiment, images were collected in the differential interference contrast mode every 30 s for 16 h. Three random areas per sample were evaluated by counting the number of cells (13 ± 3) and MBs (55 ± 5) that were used to calculate the MB/cell uptake ratio.

### MyEnd^+/+^ Cells Incubated with Plain MBs

To one OC, 9 ml of medium and 1 ml of cell suspension was added at a cell concentration of 3.2 × 10^6^ ml^−1^, giving a total of 3.2 × 10^6^ cells per OC or 6.4 × 10^4^ cm^−2^. The OCs with cell suspensions were then placed in the incubator horizontally. Images were taken after seeding on the same day and the day after.

Two days after cell seeding, the medium was removed from the OC and replaced with 9 ml of fresh medium mixed with 1 ml sterile suspension of non-fluorescent MBs per OC to obtain three different MB concentrations: 10^6^, 3 × 10^6^, and 5 × 10^6^ ml^−1^ (named OC1, OC2, and OC3, respectively). Two controls were used: one OC with only cells (named OC4), and one control containing only MBs at a concentration of 5 × 10^6^ ml^−1^ (named OC5). The OCs were placed in the incubator horizontally but flipped up-side-down to allow MB/cell interactions. Images were taken directly before and 1 h after MBs were added to the cells. Thereafter, images were taken at 5 and 26 h after addition of MBs, and the cells in the OCs were evaluated by light microscopy.

## Results

### RAW264.7 Cells Incubated with Plain MBs

**Figure 2:**
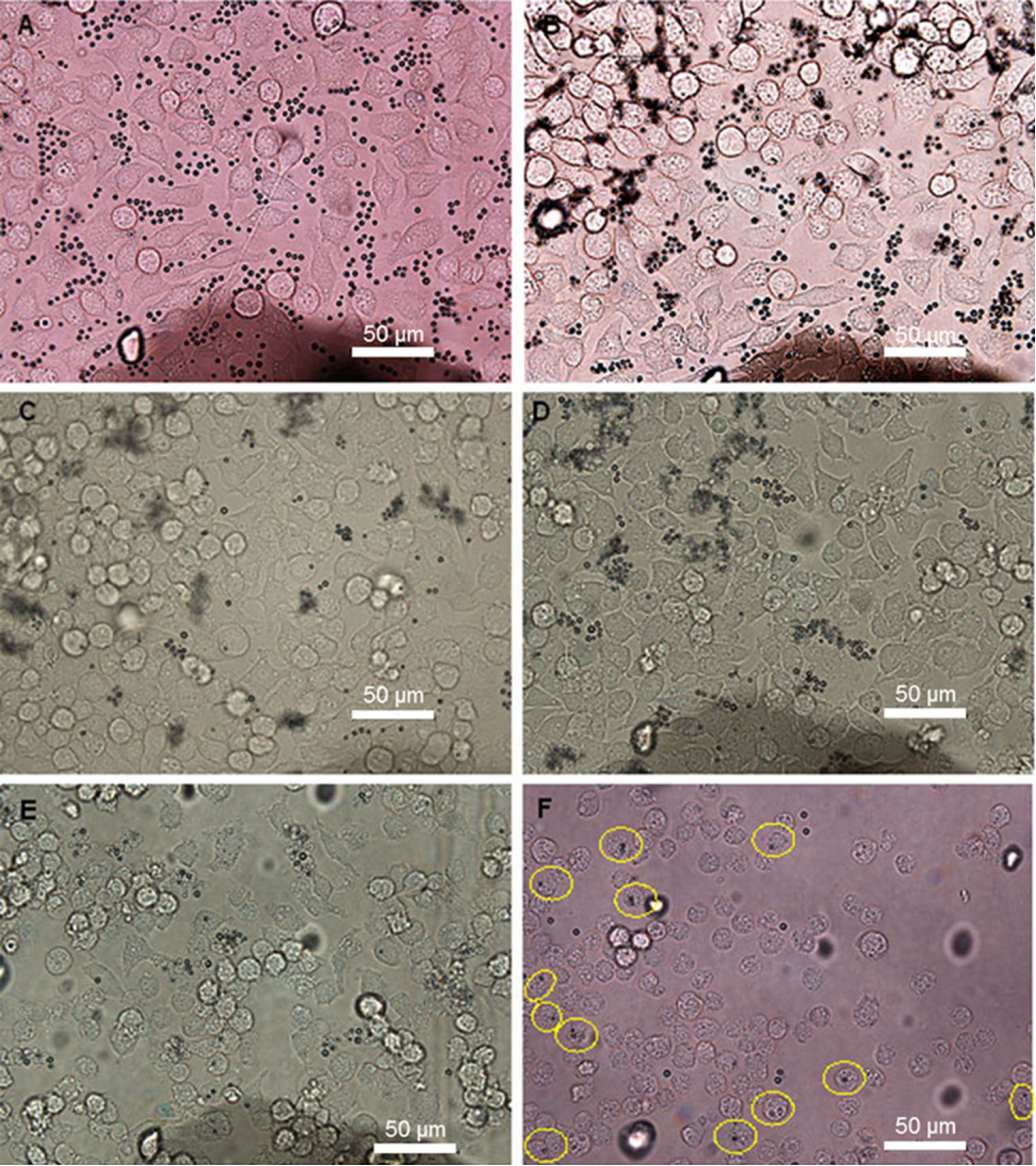
Light microscope images (40×) of macrophages in OC2 (MB concentration 10^6^ mL^−1^). Images were taken: (a) directly after MB addition; (b) 1 h after MB addition; (c) 19 h after MB addition; (d) 26 h after MB addition; (e) 48 h after MB addition before change of medium and (f) after change of medium. Yellow rings show cells with internalized MBs.

To verify the MB interactions with macrophages visually, several sets of images (40× magnification) were taken in the same ROI (Fig. 2) at different time-points after incubation with MBs. Images were taken just before addition of MBs and at 1, 19, 26, and 48 h after the addition of plain MBs. Cells were also imaged after the medium was changed at 48 h.

One hour after the addition of MBs, no changes were seen compared with images taken directly after addition of MBs. Images taken at 19 h after MB addition showed no difference compared with images taken at 26 h. The number of cells increased in the first 26 h after incubation with MBs and decreased thereafter. At 48 h, the number of both cells and MBs decreased, and more empty areas were seen in OptiCells™ (OCs 1–3). The cells exhibited a more rounded morphology, and the same phenomenon was observed in control OC4 containing only cells. A decrease in MB concentration was seen in images taken at 19 h and later, whereas MB concentration remained relatively constant in the control OC5 with only MBs (data not shown).

**Figure 3:**
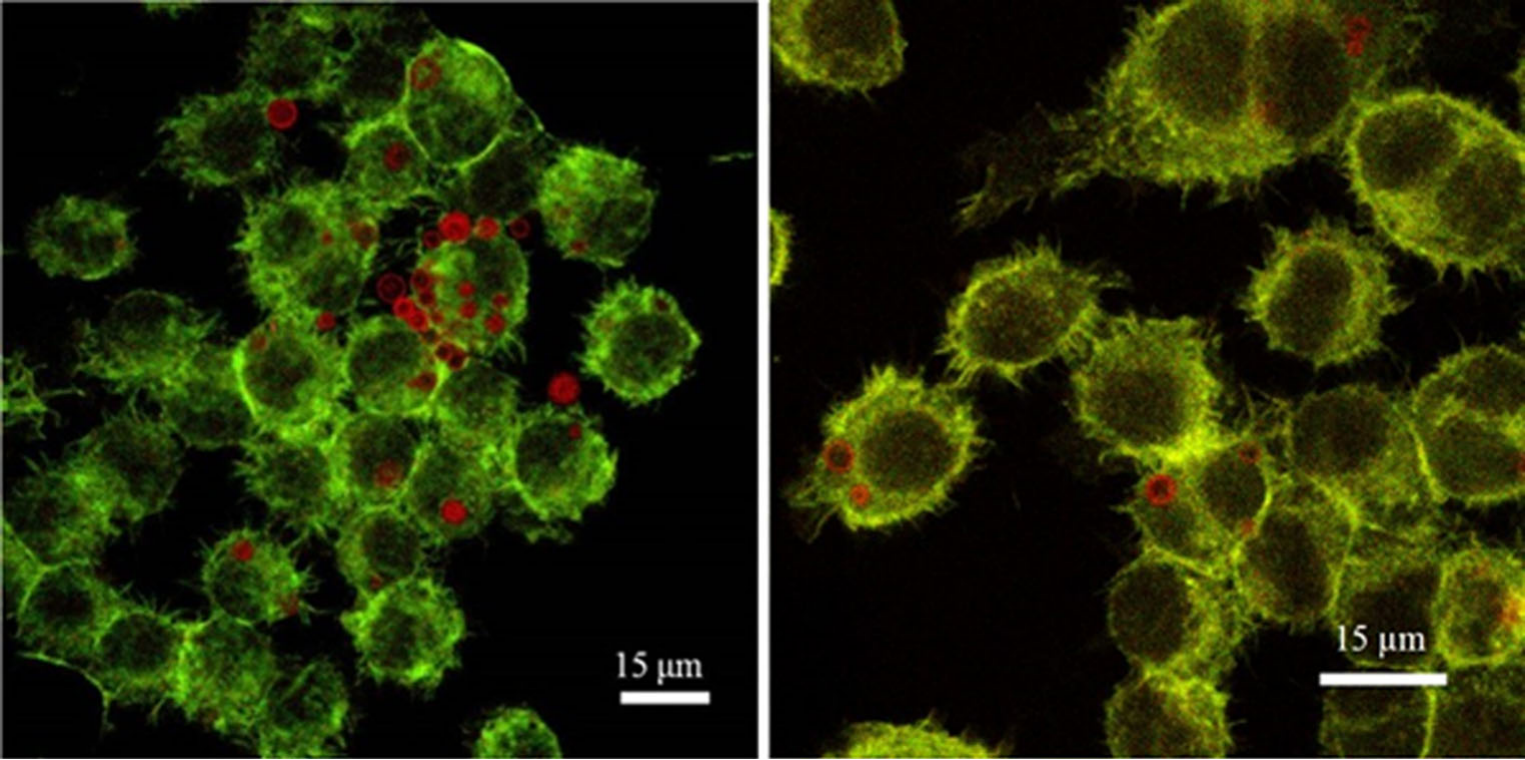
Two different CLSM images (60×, zoom factor 1and 1.5) of FITC–phalloidin-labelled RAW264.7 cells incubated for 6 h with plain rhodamine-labelled MBs. Some plain MBs were taken up by cells without being degraded after 6 h.

In a second experiment, we used CLSM to examine the behavior of macrophages incubated with plain rhodamine-labelled MBs as a further control. Three time points, 6, 24, and 72 h, were evaluated, and uptake was observed at all times. RAW264.7 macrophages have been shown to internalize these MBs after 8 h, but not after 4 h.^42^ Thus, an uptake of plain MBs was observed after incubation with macrophages longer than 4 h and was noted at 6, 8, 24, and 72 h, however not reliably quantifiable (Fig. 3). We carried out calculation of plain MB uptake after 6 h as the shortest observed incubation time with an uptake, and 72 h as the longest observation time we applied. In 4 separate calculations of 7, 19, 25, and 23 cells after 6 h and 81, 61, 60, and 60 cells after 72 h, we found an average (SD) uptake of 0.69 (0.21) MBs per cell after 6 h and 0.17 (0.04) MBs after 72 h.

### RAW264.7 Incubated with Plain and SPION-Coated MBs and Time Lapse Study

To identify the minimum time needed for internalization of type A SPION–MBs by RAW264.7 cells, we performed a time-lapse experiment in which we collected images every 30 s. The first events of uptake occurred after 25–75 min (images not shown). MBs filled with air might have somewhat different physical properties in relation to cells than the MBs filled with PBS used in this experiment, but the results of these experiments might still indicate when the uptake process was initiated.

**Figure 4:**
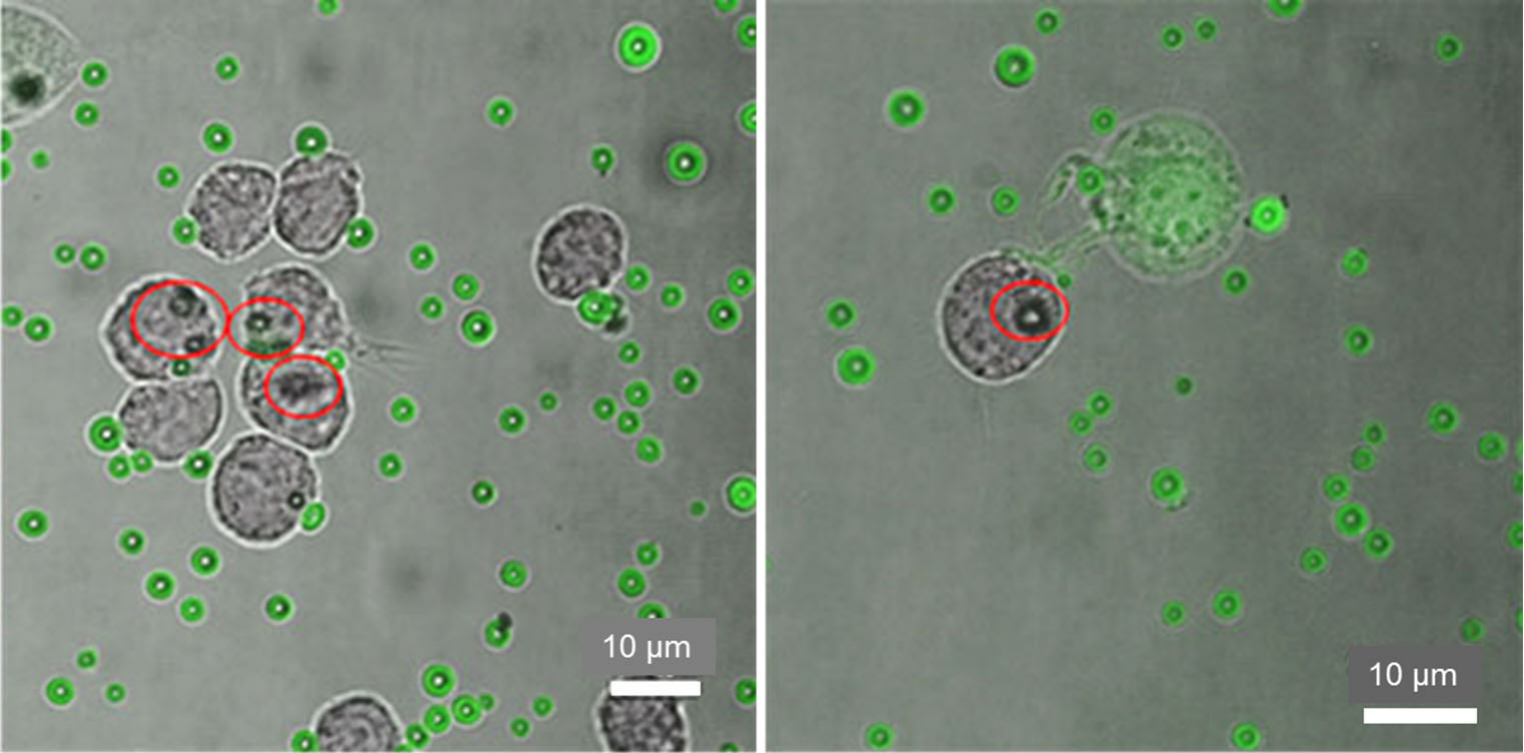
Two different merged light microscopy and CLSM images (60×, zoom factor 1and 1.3) of macrophages incubated with FITC-labelled type A MBs for 2 h (20 MBs per cell). Red rings represent MBs at different focal planes with respect to the others.

**Figure 5:**
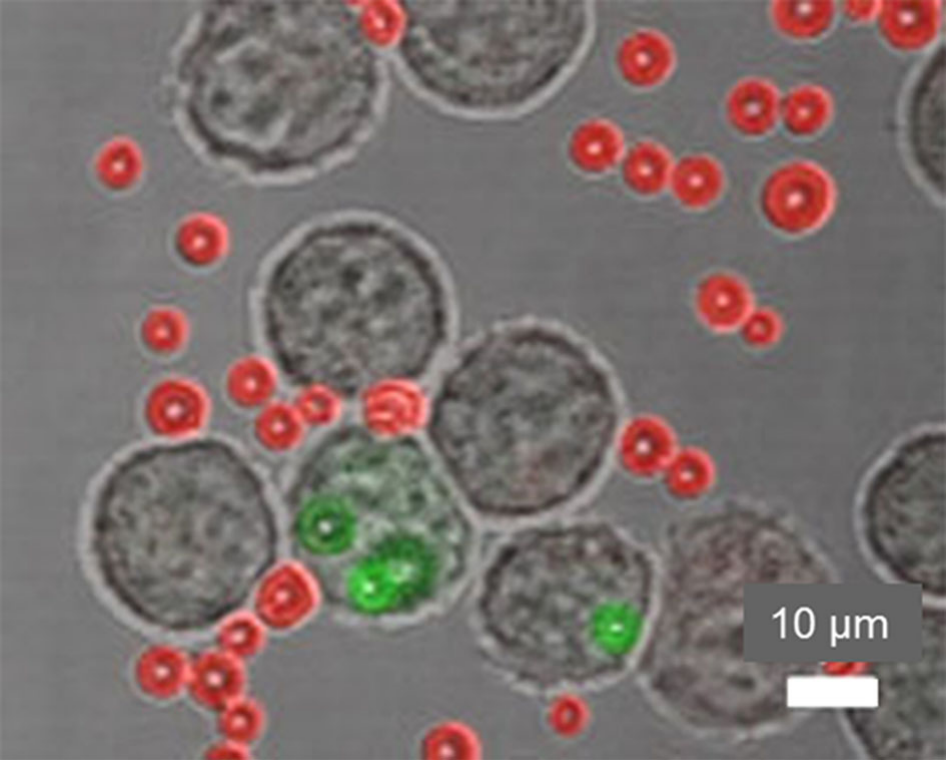
Merged light microscopy and CLSM images (60×, zoom factor 1.8) of type A MBs taken up by cells after 2 h. FITC fluorescence was quenched by TB. MBs inside the cells were not quenched and remained green, and MBs outside cells were quenched and appear red.

**Figure 6:**
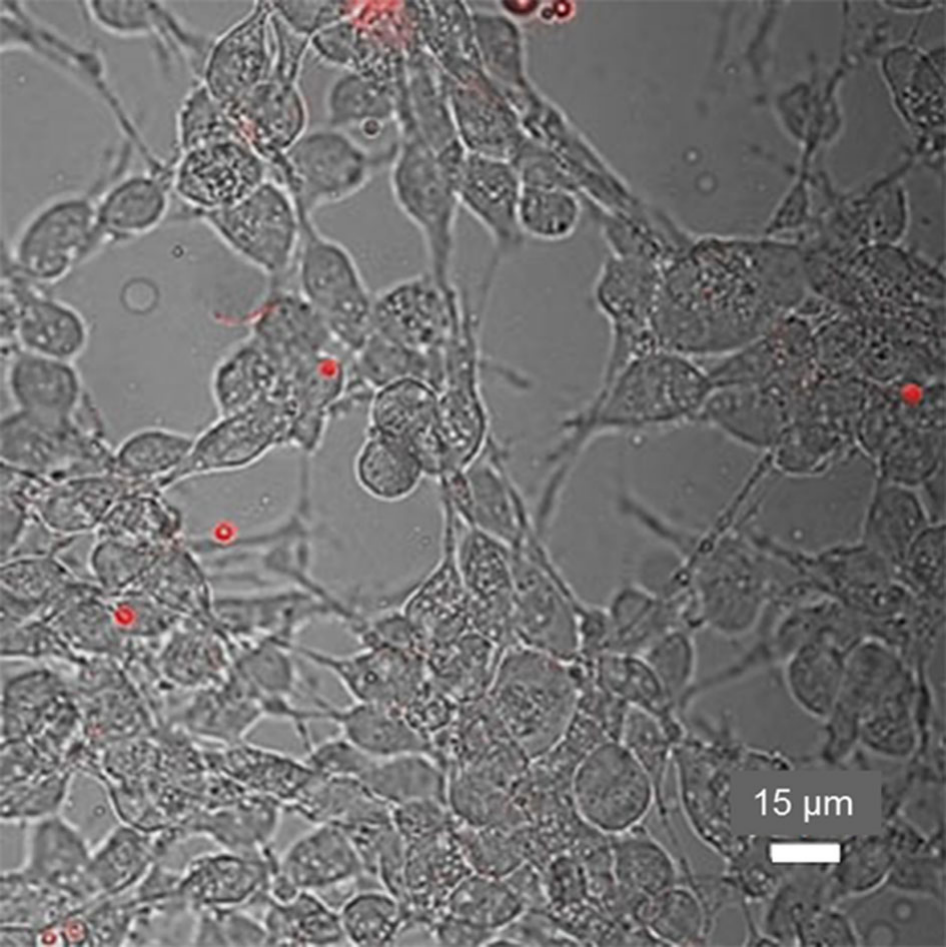
Merged light microscopy and CLSM images (40×) of TB-treated MyEnd^+/^+ cells incubated with type A MBs. FITC-quenched MBs appear red and indicate that no uptake occurred.

The faster and higher level of uptake of type A SPION–MBs by macrophages prompted us to investigate the cellular interactions of this type of MB in the following experiments (Figs. 4, 5, and 6). CLSM visualization of FITC- and SPION–FITC-labelled MBs (types A and B) with macrophages showed little or no co-localization between the MBs and the cells within 30 min.

**Figure 7:**
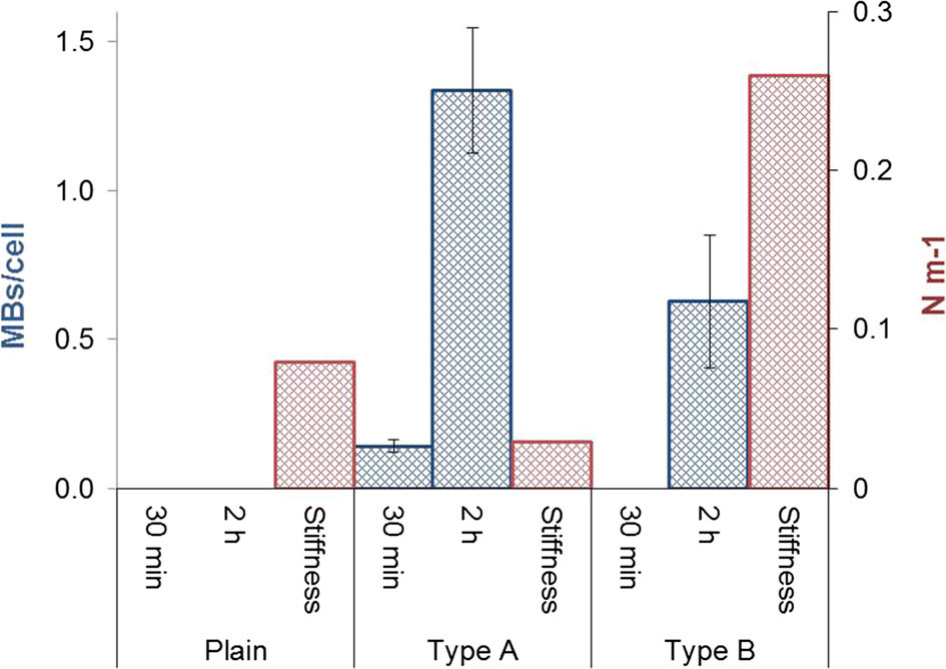
Uptake (MBs per cell) of plain and types A and B MBs by macrophages after 30 min and 2 h (blue bars). Five to nine areas were counted per sample. The stiffness of the MBs is presented on the right-hand axis (red bars). Bars represent the mean ± SD.

Extending the incubation time between macrophages and MBs to 2 h allowed us to recognize differences in behavior between MBs with and without SPIONs (Fig. 7). Co-localization with cells and internalization of type A MBs after 2 h are shown in Figs. 4 and 5.

Incubation of macrophages with plain and types A and B MBs showed some uptake of type A MBs. After 30 min, about 0.14 type A MBs per cell were internalized, and after 2 h, about 1.34 MBs per cell were engulfed. Regarding type B, about 0.63 MBs per cell were found inside the cell after 2 h yielding an uptake ratio of 2.1:1 in favor of MB type A at 2 h exposure time. No uptake of plain MBs was observed after 30 min or after 2 h.

The resulting MB uptake within 2 h is summarized in Fig. 7.

### MyEnd^+/+^ Cells Incubated with Plain or Type A-SPION–MBs

**Figure 8:**
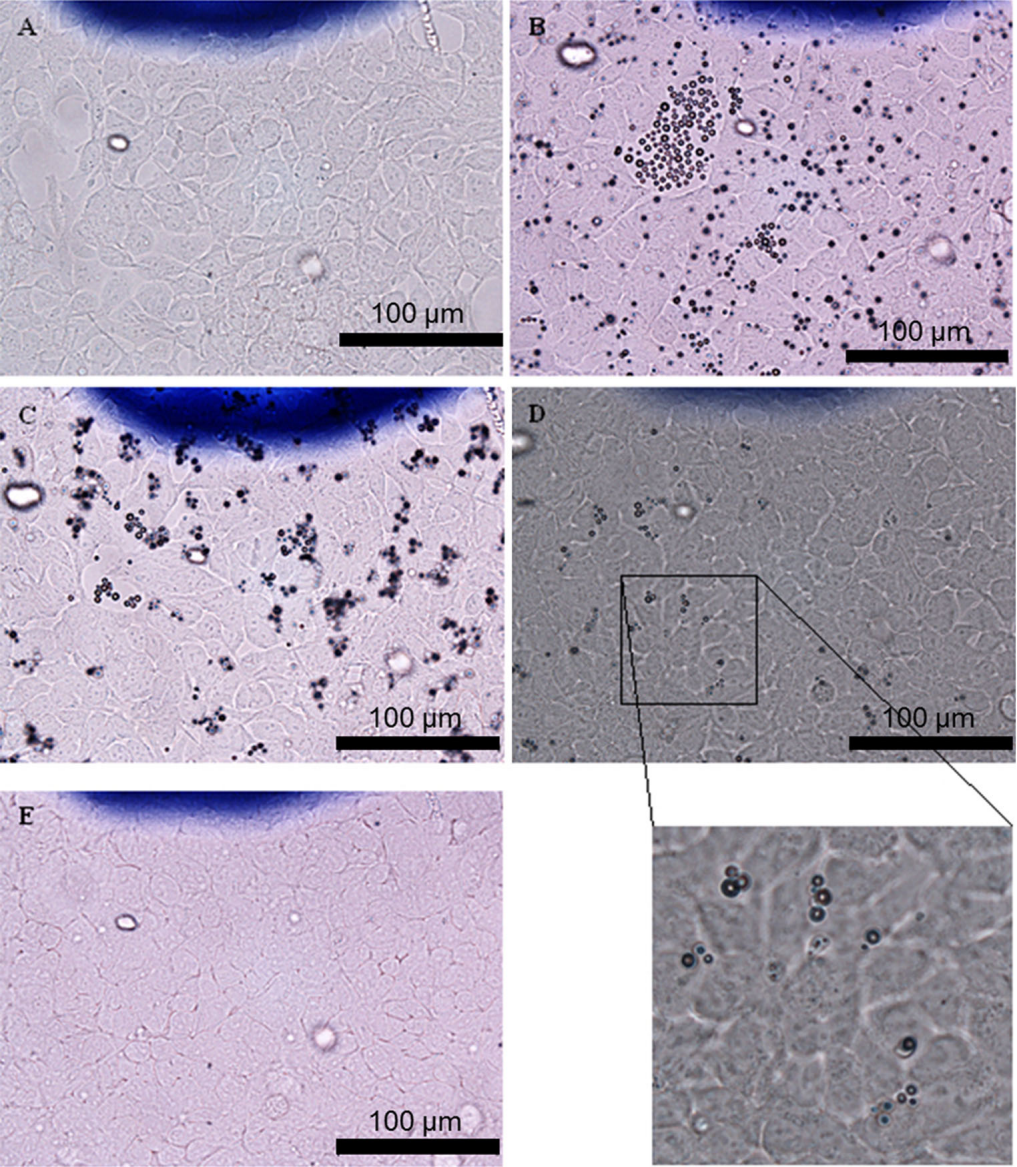
Light microscope images (40×) of MyEnd^+/+^ cells. (a) 2 days after seeding; (b) 1 h after addition of plain MBs; (c) 5 h after addition of plain MBs; (d) 26 h after addition of plain MBs, including a higher-magnification image; and (e) after washing.

Two days after seeding, cells were confluent and plain MBs mixed with fresh medium were added (Figs. 8a and 8b). Five hours after addition of the plain MBs at a concentration 10^6^ ml^−1^, MBs appeared clustered together on the cell surface (Fig. 8c). Images were then taken in the same ROI after the cell culture medium was changed to normal medium without MBs after 26 h of incubation with MBs. At this time, virtually no MBs remained in the OCs, which suggested that the adherence of MBs to MyEnd^+/+^ cells was low (Fig. 8d), as would be expected for endothelial cells.

We next examined the bio-interface between MyEnd^+/+^ cells and type A SPION–MBs. Two days after incubation, washing and TB treatment (Fig. 6), we observed low adherence and no uptake of the MBs. In this case, the behavior of type A MBs did not differ from that of plain MBs.

## Discussion

### RAW264.7 Cells

When cells are incubated in the OCs a decrease in concentrations can be observed. The decrease in MB concentration, seen in the later time-points, is probably caused by engulfment of the MBs by macrophages. Internalized MBs and MBs attached on the cell surfaces are seen at 19, 26, and 48 h after incubation with MBs and after the change in medium. More MBs are found after incubation in OC3 than in OCs 1, and 2, and these differences correspond to differences in MB concentration between OCs (data not shown). A few MBs were seen on the bottom of OCs 1–3 and OC5, possibly because of leakage of the MB shell, which reduced the floating ability of the MBs.

From the experiments made in Ibidi^®^-slides and evaluated by CLSM we concluded that an uptake of MBs occurred within 4–6 h of incubation with macrophages (Fig. 3). Most MBs were internalized within this time frame and remained intact. As others have shown intact internalized microbubbles to be acoustically active,^34^ their US signal in tissue may be detected after clearance of circulating MBs from the blood pool.

The incubation of macrophages with plain, types A and B MBs showed uptake of type A MBs already after 30 min, which was ten-folded after 2 h, while the type B MBs were found inside the cells only after 2 h. Thus, after 2 h, type A MBs were internalized at a 113% higher rate compared with the uptake of type B. No uptake of plain MBs was observed after any of the two time-points. CLSM visualization of FITC- and SPION–FITC-labelled MBs (types A and B) with macrophages showed little or no co-localization between the MBs and the cells within 30 min. Extending the incubation time between macrophages and MBs to 2 h allowed us to recognize differences in behavior between MBs with and without SPIONs (Fig. 7). The faster and higher level of uptake of type A SPION–MBs by macrophages prompted us to investigate the cellular interactions of mainly this type of MB further in the following experiments (Figs. 4, 5, 6).

To prove that SPION–MBs were internalized by the macrophages, TB (0.1 mg ml^−1^) in PBS was used to quench the FITC-signal from the non-internalized MBs. It has previously been shown that TB can quench the fluorescence of FITC-labelled compounds, ^54^ and cannot pass through the intact membranes of viable cells because of its negative charge; therefore, it is unable to quench the fluorescence signal from intracellular MBs. We found that SPION–FITC-labelled MBs outside macrophages lost their green fluorescence and acquired red fluorescence, whereas SPION–FITC-labelled MBs inside cells maintained their green fluorescence (Fig. 5). Our data showing uptake of MBs indicated that macrophages recognized MBs coated with SPIONs as exogenous and internalized them faster than uncoated MBs. This is in agreement with a previous study that reported internalization of uncoated MBs at 4–8 h.^52^ Co-localization with cells and internalization of type A MBs after 2 h are shown in Figs. 4 and 5.

Macrophage uptake of type B MBs after 2 h was also confirmed* in vivo* by Barrefelt* et al*. using histopathology.^4^ There is an interest for* in vitro* adhesion and absorption profiles and mechanism of internalization for particles smaller than 0.5 μm. In our case we analyzed the uptake of micron-size bubbles with an average diameter of 3.5 μm, which are internalized by macrophages, professional phagocytic cells, but not by the endothelial cells. Due to these evidences, we exclude both clathrin- and caveolae-mediated endocytosis that is usually used by many types of cells to internalize nanoscale materials and pinocytosis used to absorb fluids. In addition, literature sustains this behaviour, since particles larger than 0.5 μm have been known to enter phagocytic cells* via* phagocytosis pathways.^11^,^45^ Phagocytosis by macrophages is also known to be driven by physical properties, such as the rigidity or stiffness of the target surfaces, among other phagocytic signals such as immunoglobulin G opsonization, lack of “the marker of self” CD47 signals, and others.^57^ The stiffness of MBs has been tested by Poehlmann* et al*. and is expressed in N m^−1^ in a force–deformation curve.^49^ Type A MB showed an average stiffness of 0.03 N m^−1^, followed by 0.08 N m^−1^ for plain, and 0.26 N m^−1^ for type B (Fig. 7). In contrast to the data published by Poehlmann* et al*.^49^ our data suggested that the MB rigidity or stiffness might not have been the only factor influencing the MB-uptake in our experiments and may be relevant considering only when comparing the type A and B MBs. Factors such as the surface charge (neutral in these cases), shape, or SPION-related factors, e.g. surface ruggedness, might explain the differences in uptake speed between plain and SPION-labelled MBs. The importance of surface charge and shape for nanoparticle uptake in stem cells has been demonstrated by Chen* et al*.^12^ and phagocytosis by liver macrophages (Kupffer cells) increase with highly positively or negatively charged nanoparticles.^65^ Also the MBs micrometer size seems to be preferred by phagocytic cells such as macrophages.^17^ The interaction of contrast agents with cells is related to the formation of the so-called “protein corona” coating, which develops upon MB exposure to biological fluids such as plasma or serum.^37^,^62^ Among plain and type B tested MBs after incubation in cell culture medium with 10% serum, albumin has been shown by Wan* et al*. to be the abundant protein in the protein corona for several different contrast agents.^62^ The protein composition seems to be driven by surface curvature more than surface charge. Albumin is known to be a suppressor of phagocytosis, and it has been found that liposome uptake by retinal epithelial cells is reduced more than two times and by murine monocyte-macrophage cell line RAW 264.7 by three times in the presence of 5% fetal calf serum.^44^ Although presence of serum might have reduced uptake of our differently modified MBs, it should better mimic an* in vivo* situation than serum free cell culture media. Iron oxide nanoparticles may trigger an immunogenic response of the macrophages* via* opsonins as complement and immunoglobulins and receptor-driven phagocytosis,^46^ thereby explaining a more vivid uptake of type A and type B MBs.

### MyEnd^+/+^ Cells

There were no indications of uptake of neither plain nor type A MB by the MyEnd^+/+^-cells. A small amount of surface adherence could be noticed but nothing that was unexpected for this cell type. The difference between medium volumes of the imaging platforms (OC, 10 ml and Ibidi^®^ slide, 1.7 µl) used in the two experimental setups for plain and type A MBs has been considered of small impact since the MB:cell ratios have been kept in the same orders of magnitude in both setups.

Recently, increasing interest has been seen in targeting MBs and other micro-sized particles for drug and gene delivery for therapeutics, and for specific molecular imaging of cancer and cardiovascular diseases.^10^,^30^,^35^,^60^,^64^,^66^,^67^ Because none of the MBs evaluated here specifically target endothelial cells, our future efforts will focus on modifying MBs in order to investigate the specific targeting of activated and inflamed cell models. With the aim of developing targeted molecular imaging probes, we plan to use antibodies against surface receptors or protein ligands to facilitate greater adhesion and uptake by these cells.

## Conclusions

Macrophages have a natural ability for engulfing foreign material. In our study microbubbles externally labelled with SPIONs (type A) are shown to be internalized 2.1 times more efficiently than the internally SPION-labelled microbubbles (type B) after 2 h. Microbubbles without SPIONs (plain) are not internalized by the macrophages after 2 h indicating that the different physical properties of the MBs, e.g. SPION labelling, could have an effect on the cellular uptake.

The experiments using endothelial cells showed no significant uptake or adhesion of plain, unmodified MBs, which did not adhere strongly enough to withstand washing. Endothelial cells do not engulf foreign material to the same extent as macrophages; therefore, endothelial cells are not expected to internalize MBs. Endothelial cell models become more relevant when stimulated to overexpress certain adhesion molecules in order to mimic inflamed tissue. More adhesion of the MBs to the cell surface can be expected, especially if the MBs also carry targeting moieties for these adhesion molecules. This will be of interest in the future evaluation of these systems.

## References

[1] Adamson RH, Curry FE, Adamson G, Liu B, Jiang Y, Aktories K, Barth H, Daigeler A, Golenhofen N, Ness W, Drenckhahn D (2002). Rho and rho kinase modulation of barrier properties: cultured endothelial cells and intact microvessels of rats and mice. J. Physiol..

[2] Bala G and Cosyns B. Recent advances in visualizing vulnerable plaque: focus on noninvasive molecular imaging. *Curr*. *Cardiol. Rep.* 16, 2014. 10.1007/s11886-014-0520-5 25059464

[3] Balkwill F, Mantovani A (2001). Inflammation and cancer: back to Virchow?. Lancet.

[4] Barrefelt A, Paradossi G, Asem H, Margheritelli S, Saghafian M, Oddo L, Muhammed M, Aspelin P, Hassan M, Brismar TB (2014). Dynamic MR imaging, biodistribution and pharmacokinetics of polymer shelled microbubbles containing SPION. Nano.

[5] Benfer M, Kissel T (2012). Cellular uptake mechanism and knockdown activity of siRNA-loaded biodegradable DEAPA-PVA-g-PLGA nanoparticles. Eur. J. Pharm. Biopharm..

[6] Briley-Saebo KC, Shaw PX, Mulder WJM, Choi S-H, Vucic E, Aguinaldo JGS, Witztum JL, Fuster V, Tsimikas S, Fayad ZA (2008). Targeted molecular probes for imaging atherosclerotic lesions with magnetic resonance using antibodies that recognize oxidation-specific epitopes. Circulation.

[7] Brismar TB, Grishenkov D, Gustafsson B, Harmark J, Barrefelt A, Kothapalli S, Margheritelli S, Oddo L, Caidahl K, Hebert H, Paradossi G (2012). Magnetite nanoparticles can be coupled to microbubbles to support multimodal imaging. Biomacromolecules.

[8] Cai XW, Yang F, Gu N (2012). Applications of magnetic microbubbles for theranostics. Theranostics.

[9] Cavalieri F, El Hamassi A, Chiessi E, Paradossi G (2005). Stable polymeric microballoons as multifunctional device for biomedical uses: synthesis and characterization. Langmuir.

[10] Cerroni B, Chiessi E, Margheritelli S, Oddo L, Paradossi G (2011). Polymer shelled microparticles for a targeted doxorubicin delivery in cancer therapy. Biomacromolecules.

[11] Champion JA, Walker A, Mitragotri S (2008). Role of particle size in phagocytosis of polymeric microspheres. Pharm. Res..

[12] Chen F, Ma M, Wang JX, Wang F, Chern SX, Zhao ER, Jhunjhunwala A, Darmadi S, Chen HR, Jokerst JV (2017). Exosome-like silica nanoparticles: a novel ultrasound contrast agent for stem cell imaging. Nanoscale.

[13] Coussens LM, Werb Z (2002). Inflammation and cancer. Nature.

[14] Dimastromatteo J, Broisat A, Perret P, Ahmadi M, Boturyn D, Dumy P, Fagret D, Riou LM, Ghezzi C (2013). In vivo molecular imaging of atherosclerotic lesions in ApoE(-/-) mice using VCAM-1-specific, Tc-99 m-labeled peptidic sequences. J. Nucl. Med..

[15] Eckert J, Schmidt M, Magedanz A, Voigtlander T, Schmermund A (2015). Coronary CT angiography in managing atherosclerosis. Int. J. Mol. Sci..

[16] Fayad ZA (2009). Cardiovascular molecular imaging. Arterioscler. Throm. Vasc..

[17] Frohlich E (2012). The role of surface charge in cellular uptake and cytotoxicity of medical nanoparticles. Int. J. Nanomed..

[18] Fytianos K, Rodriguez-Lorenzo L, Clift MJ, Blank F, Vanhecke D, von Garnier C, Petri-Fink A, Rothen-Rutishauser B (2015). Uptake efficiency of surface modified gold nanoparticles does not correlate with functional changes and cytokine secretion in human dendritic cells* in vitro*. Nanomed-Nanotechnol..

[19] Golenhofen N, Ness W, Wawrousek EF, Drenckhahn D (2002). Expression and induction of the stress protein alpha-B-crystallin in vascular endothelial cells. Histochem. Cell Biol..

[20] Guo HZ, Jiang ZQ, Song S, Dai TT, Wang XY, Sun K, Zhou GD, Dou HJ (2016). Structural regulation of self-assembled iron oxide/polymer microbubbles towards performance-tunable magnetic resonance/ultrasonic dual imaging agents. J. Colloid Interface Sci..

[21] Hansson GK (2005). Mechanisms of disease—inflammation, atherosclerosis, and coronary artery disease. New Engl. J. Med..

[22] Harmark J, Hebert H, Koeck PJB (2016). Shell thickness determination of polymer-shelled microbubbles using transmission electron microscopy. Micron.

[23] Harmark J, Larsson MK, Razuvajev A, Koeck PJB, Paradossi G, Brodin LA, Caidahl K, Hebert H, Bjallmark A (2015). Investigation of the elimination process of a multimodal polymer-shelled contrast agent in rats using ultrasound and transmission electron microscopy. Biomed. Spectrosc. Imaging.

[24] He W, Yang F, Wu YH, Wen S, Chen P, Zhang Y, Gu N (2012). Microbubbles with surface coated by superparamagnetic iron oxide nanoparticles. Mater. Lett..

[25] Ino JM, Chevallier P, Letourneur D, Mantovani D, Le Visage C (2013). Plasma functionalization of poly(vinyl alcohol) hydrogel for cell adhesion enhancement. Biomatter.

[26] Jaffer FA, Libby P, Weissleder R (2009). Optical and multimodality molecular imaging insights into atherosclerosis. Arterioscler. Thromb. Vasc. Biol..

[27] Jokerst JV, Khademi C, Gambhir SS (2013). Intracellular aggregation of multimodal silica nanoparticles for ultrasound-guided stem cell implantation. Sci. Transl. Med..

[28] Kothapalli S, Daeichin V, Mastik F, Brodin LA, Janerot-Sjoberg B, Paradossi G, de Jong N, Grishenkov D (2015). Unique pumping-out fracturing mechanism of a polymer-shelled contrast agent: an acoustic characterization and optical visualization. IEEE T Ultrason. Ferr..

[29] Kothapalli S, Oddo L, Paradossi G, Brodin LA, Grishenkov D (2014). Assessment of the viscoelastic and oscillation properties of a nano-engineered multimodality contrast agent. Ultrasound Med. Biol..

[30] Kupal SG, Cerroni B, Ghugare SV, Chiessi E, Paradossi G (2012). Biointerface properties of core-shell poly(vinyl alcohol)-hyaluronic acid microgels based on chemoselective chemistry. Biomacromolecules.

[31] Larsson MK, Larsson M, Nowak G, Paradossi G, Brodin LA, Sjoberg BJ, Caidahl K, Bjallmark A (2014). Endocardial border delineation capability of a novel multimodal polymer-shelled contrast agent. Cardiovasc. Ultrasound.

[32] Larsson M, Larsson M, Oddo L, Margheritelli S, Paradossi G, Nowak J, Brodin LA, Caidahl K, Bjallmark A (2013). Visualization of multimodal polymer-shelled contrast agents using ultrasound contrast sequences: an experimental study in a tissue mimicking flow phantom. Cardiovasc. Ultrasound.

[33] Libby P (2002). Inflammation in atherosclerosis. Nature.

[34] Lindner JR, Dayton PA, Coggins MP, Ley K, Song J, Ferrara K, Kaul S (2000). Noninvasive imaging of inflammation by ultrasound detection of phagocytosed microbubbles. Circulation.

[35] Liu J, Zhang P, Liu P, Zhao Y, Gao S, Tan K, Liu Z (2012). Endothelial adhesion of targeted microbubbles in both small and great vessels using ultrasound radiation force. Mol Imaging.

[36] Louie AY (2010). Multimodality imaging probes: design and challenges. Chem. Rev..

[37] Mahmoudi M, Sant S, Wang B, Laurent S, Sen T (2011). Superparamagnetic iron oxide nanoparticles (SPIONs): development, surface modification and applications in chemotherapy. Adv Drug Deliv Rev.

[38] Manabe I (2011). Chronic inflammation links cardiovascular, metabolic and renal diseases. Circ. J..

[39] Mickova A, Buzgo M, Benada O, Rampichova M, Fisar Z, Filova E, Tesarova M, Lukas D, Amler E (2012). Core/shell nanofibers with embedded liposomes as a drug delivery system. Biomacromolecules.

[40] Mikhaylova M, Kim DK, Berry CC, Zagorodni A, Toprak M, Curtis ASG, Muhammed M (2004). BSA immobilization on amine-functionalized superparamagnetic iron oxide nanoparticles. Chem. Mater..

[41] Mitsumori LM, Bhargava P, Essig M, Maki JH (2014). Magnetic resonance imaging using gadolinium-based contrast agents. Top Magn. Reson. Imaging.

[42] Mozetic P, Tortora M, Cerroni B, Paradossi G, Paradossi G, Pellegretti P, Trucco A (2010). Polymer based biointerfaces: a case study on devices for theranostics and tissue engineering. Ultrasound Contrast Agents—Targeting and Processing Methods for Theranostics.

[43] Nahrendorf M, Weissleder R (2007). Advances in cardiovascular medicine through molecular imaging. Radiologe.

[44] Niesman MR, Peyman GA, Miceli MV (1997). Liposome uptake by human retinal pigment epithelial cells in culture. Curr. Eye Res..

[45] Oh N, Park JH (2014). Endocytosis and exocytosis of nanoparticles in mammalian cells. Int. J. Nanomed..

[46] Owens DE, Peppas NA (2006). Opsonization, biodistribution, and pharmacokinetics of polymeric nanoparticles. Int. J. Pharm..

[47] Paradossi G, Cavalieri F, Chiessi E, Ponassi V, Martorana V (2002). Tailoring of physical and chemical properties of macro- and microhydrogels based on telechelic PVA. Biomacromolecules.

[48] Park JI, Jagadeesan D, Williams R, Oakden W, Chung SY, Stanisz GJ, Kumacheva E (2010). Microbubbles loaded with nanoparticles: a route to multiple imaging modalities. Acs. Nano..

[49] Poehlmann M, Grishenkov D, Kothapalli S, Harmark J, Hebert H, Philipp A, Hoeller R, Seuss M, Kuttner C, Margheritelli S, Paradossi G, Fery A (2014). On the interplay of shell structure with low- and high-frequency mechanics of multifunctional magnetic microbubbles. Soft Matter..

[50] Raschke WC, Baird S, Ralph P, Nakoinz I (1978). Functional macrophage cell lines transformed by abselon leukemia-virus. Cell.

[51] Ross R (1999). Mechanisms of disease—Atherosclerosis—An inflammatory disease. New Engl. J. Med..

[52] Rudd JHF, Hyafil F, Fayad ZA (2009). Inflammation imaging in atherosclerosis. Arterioscl. Throm. Vasc..

[53] Sciallero C, Balbi L, Paradossi G, Trucco A (2016). Magnetic resonance and ultrasound contrast imaging of polymer-shelled microbubbles loaded with iron oxide nanoparticles. Royal Soc. Open Sci..

[54] Sciallero C, Grishenkov D, Kothapalli S, Oddo L, Trucco A (2013). Acoustic characterization and contrast imaging of microbubbles encapsulated by polymeric shells coated or filled with magnetic nanoparticles. J. Acoust. Soc. Am..

[55] Shi ZL, Neoh KG, Kang ET, Shuter B, Wang SC, Poh C, Wang W (2009). (Carboxymethyl)chitosan-modified superparamagnetic iron oxide nanoparticles for magnetic resonance imaging of stem cells. Acs Appl. Mater. Interfaces.

[56] Song S, Guo HZ, Jiang ZQ, Jin YQ, Wu Y, An X, Zhang ZF, Sun K, Dou HJ (2015). Self-assembled microbubbles as contrast agents for ultrasound/magnetic resonance dual-modality imaging. Acta Biomaterialia.

[57] Sosale NG, Spinler KR, Alvey C, Discher DE (2015). Macrophage engulfment of a cell or nanoparticle is regulated by unavoidable opsonization, a species-specific ‘Marker of Self’ CD47, and target physical properties. Curr. Opin. Immunol..

[58] Tachibana Y, Enmi J, Mahara A, Iida H, Yamaoka T (2010). Design and characterization of a polymeric MRI contrast agent based on PVA for in vivo living-cell tracking. Contrast Media Mol..

[59] Tang TY, Muller KH, Graves MJ, Li ZY, Walsh SR, Young V, Sadat U, Howarth SPS, Gillard JH (2009). Iron oxide particles for atheroma imaging. Arterioscler. Throm. Vasc..

[60] Villa R, Cerroni B, Vigano L, Margheritelli S, Abolafio G, Oddo L, Paradossi G, Zaffaroni N (2013). Targeted doxorubicin delivery by chitosan-galactosylated modified polymer microbubbles to hepatocarcinoma cells. Colloid Surf. B..

[61] Villanueva FS (2008). Molecular imaging of cardiovascular disease using ultrasound. J. Nucl. Cardiol..

[62] Wan S, Egri G, Oddo L, Cerroni B, Dahne L, Paradossi G, Salvati A, Lynch I, Dawson KA, Monopoli MP (2016). Biological in situ characterization of polymeric microbubble contrast agents. Int. J. Biochem. Cell Biol..

[63] Wildgruber M, Swirski FK, Zernecke A (2013). Molecular Imaging of Inflammation in Atherosclerosis. Theranostics.

[64] Wu J, Leong-Poi H, Bin J, Yang L, Liao Y, Liu Y, Cai J, Xie J, Liu Y (2011). Efficacy of contrast-enhanced US and magnetic microbubbles targeted to vascular cell adhesion molecule-1 for molecular imaging of atherosclerosis. Radiology.

[65] Xiao K, Li Y, Luo J, Lee JS, Xiao W, Gonik AM, Agarwal RG, Lam KS (2011). The effect of surface charge on in vivo biodistribution of PEG-oligocholic acid based micellar nanoparticles. Biomaterials.

[66] Xie A, Belcik T, Qi Y, Morgan TK, Champaneri SA, Taylor S, Davidson BP, Zhao Y, Klibanov AL, Kuliszewski MA, Leong-Poi H, Ammi A, Lindner JR (2012). Ultrasound-mediated vascular gene transfection by cavitation of endothelial-targeted cationic microbubbles. J. Am. Coll. Cardiol. Imaging.

[67] Yoo MK, Park IY, Kim IY, Park IK, Kwon JS, Jeong HJ, Jeong YY, Cho CS (2008). Superparamagnetic iron oxide nanoparticles coated with mannan for macrophage targeting. J. Nanosci. Nanotechnol..

